# A Primary Bone Diffuse Large B-Cell Lymphoma with Ocular Adnexal Involvement

**DOI:** 10.4274/tjh.2015.0424

**Published:** 2016-08-19

**Authors:** Rafet Eren, Ceyda Aslan, Cihan Gündoğan, Osman Yokuş, Mehmet Hilmi Doğu, Elif Suyanı

**Affiliations:** 1 İstanbul Training and Research Hospital, Clinic of Hematology, İstanbul, Turkey; 2 İstanbul Training and Research Hospital, Clinic of Nuclear Medicine, İstanbul, Turkey

**Keywords:** Primary bone lymphoma, Ocular adnexal lymphoma, Diffuse large B-cell lymphoma

## To the Editor,

Primary bone lymphomas (PBLs) [1,2] and ocular adnexal (OA) lymphomas [3,4] are rare types of extranodal lymphomas. Coexistence of these two rare entities without lymph node infiltration has not been reported previously.

A 55-year-old man presented with left hip pain without a history of trauma. His medical history and physical examination did not reveal any remarkable findings. The X-ray radiographs of the pelvis and left hip showed multiple lytic lesions. Body 18fluorodeoxyglucose positron emission tomography/computed tomography (18F-FDG PET/CT) demonstrated multiple osteolytic bone lesions in the left zygomatic bone, vertebral column, bilateral iliac bones, left caput femoris, and trochanter major of the femur with increased 18F-FDG uptake (SUVmax: 31) (Figure 1a). Tru-Cut biopsy of the caput femoris showed atypical lymphoid cells that were pancreatin (-), s-100 (-), CD138 (+), CD30 (-), CD20 (+), CD3 (-), CD5 (-), CD10 (-), bcl-6 (+), and MUM-1 (+), consistent with diffuse large B-cell lymphoma. Laboratory data were as follows: erythrocyte sedimentation rate, 37 mm/h; lactate dehydrogenase level, 405 U/L; hemoglobin, 12 g/dL; white blood cell count, 6.74x109/L; platelet count, 250x109/L; normal liver and renal function tests. Bone marrow aspirate and core biopsy were normal. A rituximab, cyclophosphamide, doxorubicine, vincristine, and prednisolone (R-CHOP) regimen was planned. However, prior to the first chemotherapy day, the patient was admitted with swelling of the eyelids, exophthalmos, proptosis, and vision loss in his left eye that developed progressively over 3 days. Magnetic resonance imaging (MRI), which was performed two months after the initial 18F-FDG PET/CT, showed an orbital mass with diameters of 36x21x38 mm eroding the superior wall of the orbita and adjacent soft tissue (Figure 1b). After the detection of orbital involvement, investigations for central nervous system (CNS) involvement were negative. Chemotherapy treatment was commenced immediately without doing a biopsy because of the patient’s vision loss. After 4 cycles of R-CHOP chemotherapy, the patient’s left hip pain, left eye swelling, exophthalmos, and proptosis resolved completely, but his vision did not improve. Control 18F-FDG PET/CT showed marked regression of bone lesions with decreased 18F-FDG uptake (SUVmax: 4.6). Orbital MRI also showed that the mass had regressed to 14x12 mm in size. After obtaining this response, 2 cycles of R-CHOP, radiotherapy to the left orbita, and two cycles of high-dose methotrexate for CNS prophylaxis were planned.

Considering the extensive lytic bone lesions and recent emergence of the OA tumor, the primary site of the disease must have been the bones in the presented case. Thus, the diagnosis can be categorized as PBL with OA involvement. An orbital mass was detected two months after diagnosis by means of MRI, but not by 18F-FDG PET/CT performed at diagnosis. It would be speculative to claim that such a large mass had arisen in a two month period. Taking into account that MRI is the gold standard imaging technique in evaluation of OA tumors [3], the orbital mass, which was probably small at the beginning, could not have been noticed on 18F-FDG PET/CT. This case emphasizes that a high suspicion index of OA involvement in PBL cases with any symptoms regarding the eyes and prompt assessment of the patients with MRI might prevent undesirable consequences.

## Ethics

Ethics Committee Approval: Not applicable; Informed Consent: Not applicable.

## Figures and Tables

**Figure 1a:**
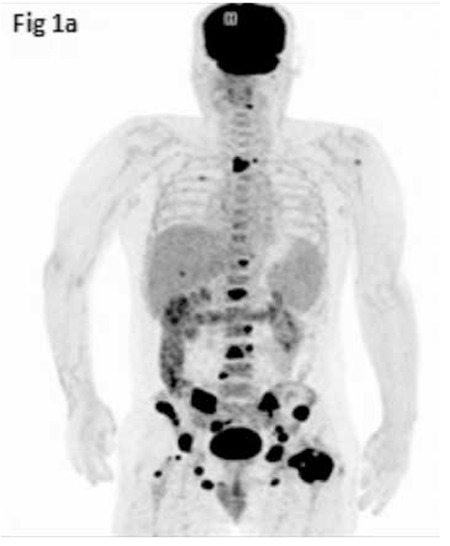
^18^Fluorodeoxyglucose positron emission tomography/computed tomography image of the patient at diagnosis.

**Figure 1b:**
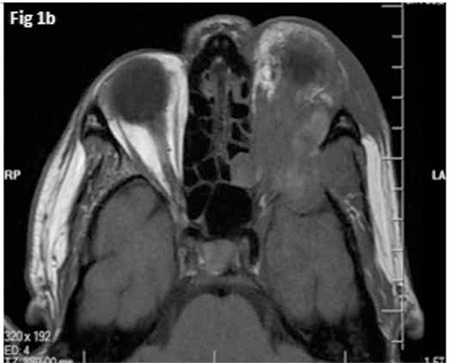
Magnetic resonance imaging image of orbital adnexal mass.
